# Developing an approach for evaluating the cardiotoxic potential of botanicals

**DOI:** 10.3389/ftox.2025.1646044

**Published:** 2025-10-30

**Authors:** Julie Krzykwa, Hemantkumar S. Chaudhari, Andre Monteiro Da Rocha, Matthias Gossmann, Peter Hoffmann, Yaser Khokhar, Nathan Meyer, Jin-Young K. Park, Robert Sprando, Ravi Vaidyanathan, Remco H. S. Westerink, Joseph C. Wu, Jeffrey Yourick, Shane R. Zhao, Constance A. Mitchell

**Affiliations:** ^1^ Health and Environmental Sciences Institute, Washington, DC, United States; ^2^ L’Oréal Research and Innovation, Clark, NJ, United States; ^3^ Frankel Cardiovascular Center Cell Regeneration Core/Internal Medicine-Cardiology, University of Michigan, Ann Arbor, MI, United States; ^4^ innoVitro GmbH, Juelich, Germany; ^5^ Consultant, Beaufort, SC, United States; ^6^ Stanford Cardiovascular Institute, Stanford University, Stanford, CA, United States; ^7^ FUJIFILM Cellular Dynamics, Inc., Madison, WI, United States; ^8^ Food and Drug Administration, Human Foods Program, Office of Food Chemical Safety Dietary Supplements and Innovation, College Park, MD, United States; ^9^ Food and Drug Administration, Human Foods Program, Office of Chemistry and Toxicology, Laurel, MD, United States; ^10^ Neurotoxicology Research Group, Division of Toxicology, Institute for Risk Assessment Sciences (IRAS), Faculty of Veterinary Medicine, Utrecht University, Utrecht, Netherlands

**Keywords:** botanical safety consortium, botanicals, cardiotoxicity, *in vitro*, new approach methods, complex mixtures

## Abstract

Botanicals (e.g., extracts derived from plants, algae, or fungi) are increasingly utilized by consumers with the hope of enhancing their health, managing symptoms, or preventing ailments; however, these products have often had limited pre-market toxicity testing. Traditional toxicity testing (e.g., rodent testing) is complicated by the nature of botanicals as complex mixtures and the potential for lot-to-lot variability in chemical constituents. Cardiotoxicity is a key area of concern, as adverse effects on the cardiovascular system can have severe consequences, and although not commonly reported, there have been reports of adverse cardiac events. New approach methodologies (NAMs) offer human-relevant, efficient, innovative, and cost-effective solutions for evaluating the cardiotoxicity of botanicals. The Botanical Safety Consortium (BSC) was established to focus on identifying suitable NAMs to screen for potential toxicities associated with these widely used products. This manuscript outlines the BSC Cardiotoxicity Working Group’s approach for evaluating NAMs for assessing the potential cardiotoxicity of botanicals. These NAMs leverage *in vitro* models, such as human-induced pluripotent stem cell-derived cardiomyocytes, and techniques like microelectrode arrays, voltage and calcium optical mapping, contractile force measurement, and mitochondrial function assays to evaluate botanical-induced effects on the cardiovascular system. Using well-characterized botanical extracts as case studies, the BSC aims to refine a toolkit for high-throughput and human-relevant cardiotoxicity screening. This foundational work supports the broader goal of improving botanical safety assessment practices and advancing the application of NAMs in regulatory toxicology.

## 1 Introduction

The use of botanical supplements, herbal remedies, and various botanical-based products has become widespread across the globe. Many consumers use these products with the hope of enhancing their health, managing symptoms, or preventing ailments. In the United States alone, the market for botanical dietary supplements reached an estimated $12.5 billion in 2023 (Smith et al., 2024). While not common, some botanical products have caused unintended adverse effects (Di Lorenzo et al., 2015). In severe cases, these effects led to permanent organ damage or even fatalities, underscoring the importance of screening botanical products to ensure their safety. Unlike other industries, where pre-market testing and assessments are standard, regulatory actions related to botanicals often occur reactively after reports of adverse events (EFSA Scientific Committee, 2009; Institute of Medicine and National Research Council of the National Academies, 2005). As such, most botanicals do not undergo specific toxicity assessments (Bent, 2008), including cardiotoxicity, leaving potential hazards unknown (Geller et al., 2015).

Assessing the safety of botanicals, particularly botanical extracts, can be difficult due to their complex and highly variable chemistry. Botanical extracts derived from plants, algae, or fungi may contain hundreds of different chemical constituents (Mitchell et al., 2022; Pyne et al., 2019). In addition, the chemical composition of a botanical extract can vary even within products sourced from the same species based on things like growing conditions, harvesting practices, manufacturing processes, and potential contamination or adulteration (Bruni and Sacchetti, 2009; Kulić et al., 2022). The inherent variation in botanical extracts makes it difficult to identify just one sample for testing in traditional animal tests with rodents or other models, as one sample may not represent the variety found in products that are available for purchase (Rombolà et al., 2020). Testing multiple samples would require considerable resources in terms of cost, time, and animal use. In addition, for cardiotoxicity, traditional methods using experimental rodent models do not always offer sufficient relevance to human biology (Beeton, 2018; Glukhov et al., 2010; Kaese and Verheule, 2012; US FDA, 2005). There is a growing demand for predictive techniques capable of efficiently evaluating cardiotoxicity in complex mixtures using rapid and resource-conscious methods. Furthermore, regulatory agencies in the United States and other jurisdictions are prioritizing the adoption of alternatives to animal testing in toxicological assessments (Reddy et al., 2023).

In response to this need, the Botanical Safety Consortium (BSC) was formed to explore and evaluate new approach methodologies (NAMs) that could help identify adverse effects induced by botanicals, including, but not limited to, potential cardiotoxicity (Mitchell et al., 2022). The BSC comprises experts from diverse fields, such as toxicology, *in vitro* methods, analytical chemistry, and pharmacognosy, across academia, industry, and government. Within the BSC, the Cardiotoxicity Working Group is focused on established *in vitro* models that may be used to screen for cardiotoxic effects. Other groups in the BSC are focused on genotoxicity, hepatotoxicity, developmental and reproductive toxicity, neurotoxicity, and dermal toxicity (Mitchell et al., 2022).

The Cardiotoxicity Working Group selected specific *in vitro* assays and botanical case studies to evaluate whether these methods are appropriate for assessing the cardiotoxic potential of botanicals as complex mixtures. This manuscript describes the approach taken by the group and includes an overview of the selected botanicals known to have cardiotoxicity potential (or lack thereof) and a discussion of relevant assays used already in single-chemical toxicity testing. These assays are being adapted to fit the complex nature of botanicals as mixtures, with the goal of advancing the understanding and safety assessment of botanicals in this crucial area of cardiotoxicity. The selected NAMs are intended to be incorporated into a larger battery of assays that could be used to comprehensively evaluate botanical safety. While currently limited to the selected screening assays, the working group recognizes the need for a broader range of tools, which can evolve over time.

**FIGURE 1:**
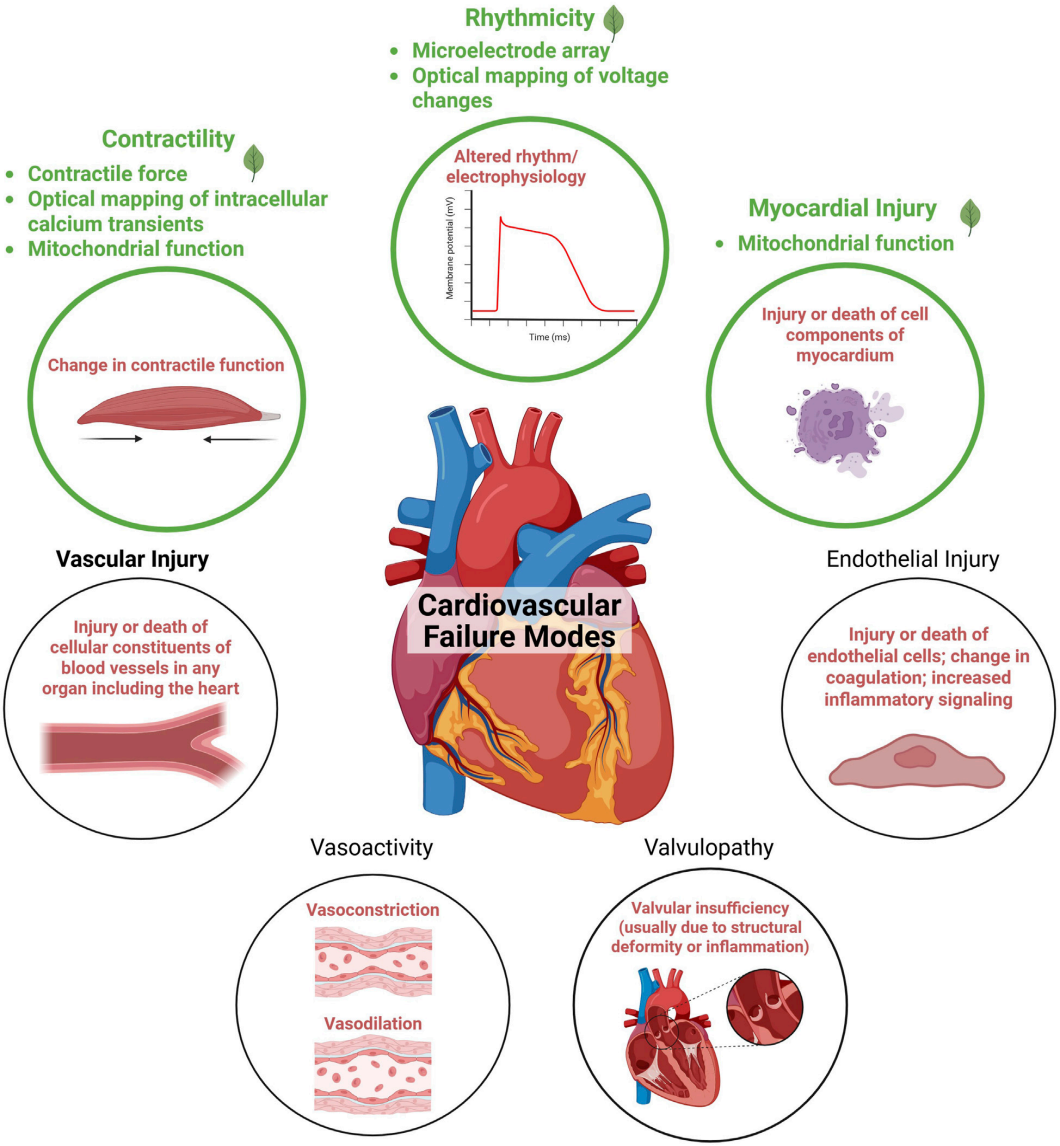
Adapted from the cardiovascular failure modes described in Berridge et al. (2024). Endpoints currently prioritized by the Botanical Safety Consortium’s cardiotoxicity working group are highlighted in green. Figure created in BioRender.

Cardiovascular toxicity encompasses adverse effects on the heart and blood vessels, which may arise through direct exposure to toxicants or indirectly via toxic metabolites or inflammatory agents. (Berridge et al., 2024). The heart’s structure and function are complex, with numerous possible targets for adverse effects from botanicals (Figure 1). Currently, the primary focus of the BSC Cardiotoxicity Working Group is on adverse effects directly impacting the heart muscle cells (cardiomyocytes). However, botanicals may also affect other types of cells in the heart (e.g., endothelial cells, fibroblasts, nodal cells, leukocytes), the cardiac vasculature, and the pulmonary and systemic vascular systems. Key cardiac functions of cardiomyocytes that can be compromised include cardiac rhythm, left-ventricular contraction, and myocardial injury. These adverse effects can have serious consequences, impacting quality of life or even posing life-threatening risks, underscoring the importance of understanding and mitigating potential cardiotoxic effects associated with botanicals.

Given the prevalence of cardiovascular disease and its significant contribution to morbidity and mortality, safeguarding cardiac health is a priority in both clinical and public health contexts. Cardiovascular disease remains the leading cause of death for men, women, and most racial and ethnic groups in the United States, accounting for approximately 20% of all deaths in the United States in 2022, with over 700,000 deaths (Martin et al., 2024). Although reports of botanical-induced cardiotoxicity are uncommon, this does not imply safety, as many botanicals on the market have not undergone cardiotoxicity evaluations. Examples below describe instances where botanicals have led to cardiotoxicity. Without routine testing, there remains a risk of potential cardiac effects. Indeed, studies have linked some botanicals to cardiotoxicity (Bhalla et al., 2015; Puschner, 2012).

The Cardiotoxicity Working Group of the BSC focuses on developing efficient screening strategies to aid in identifying botanicals with potential cardiotoxicity. Their work involves selecting and evaluating NAMs to investigate if they can accommodate botanicals as complex chemical mixtures. Using candidate botanicals chosen based on known or suspected cardiotoxicity, the working group is establishing a series of case studies to evaluate these new methodologies. This manuscript describes the approach taken by the Cardiotoxicity Working Group and includes an overview of botanicals known to have cardiotoxicity potential or no known cardiotoxicity and a discussion of relevant tools traditionally used for single-chemical toxicity testing. These tools are being adapted to fit the nature of botanicals as complex mixtures to advance the understanding and safety assessment of botanicals in this crucial area of cardiotoxicity.

## 2 Selected assays for cardiotoxicity screening

**FIGURE 2:**
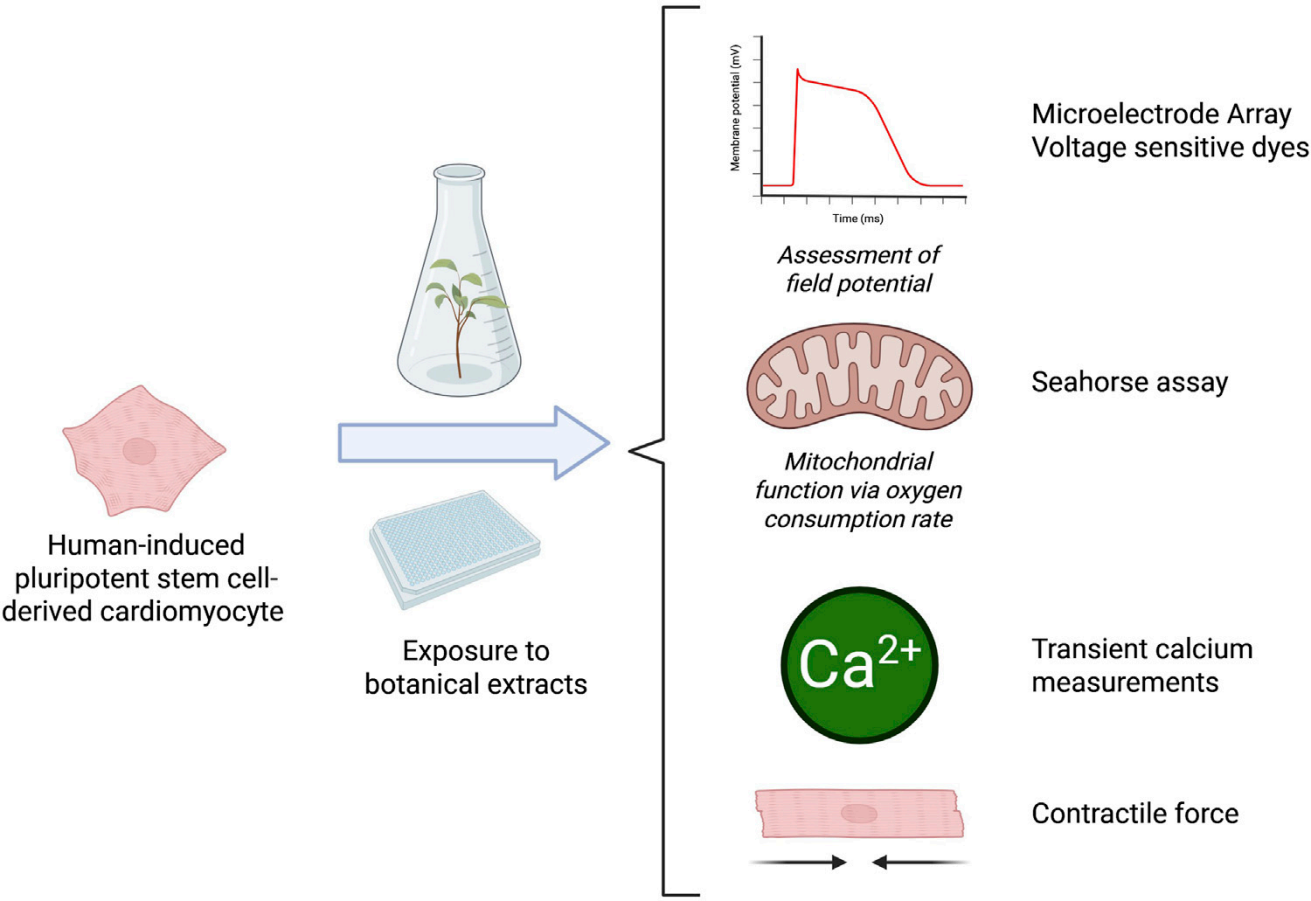
Overview of the model system and experimental setups used to determine the suitability of these assays for botanicals. Microelectrode Array (MEA) recordings can be used to assess cardiac-modulatory potential of botanical extracts including changes in several functional parameters, like beat rate, field potential duration, and spike amplitude. Mitochondrial function assessment is used to assess mitochondrial dysfunction in cardiomyocytes that could contribute to impaired ATP production, increased oxidative stress, and cell death. The optical mapping of transient calcium measurements can provide insights into calcium-handling abnormalities associated with arrhythmogenesis, contractile dysfunction, and other cardiac pathologies. The contractile force assay provides key insights into potential effects on the contractile properties of heart cells. Figure created in BioRender.

To effectively screen for botanical-induced cardiotoxicity, there is a need for a battery of assays that capture a range of mechanisms relevant to the heart. The proposal outlined here will evaluate multiple tools for their potential to screen for the cardiotoxicity of botanicals and investigate if there are considerations unique to botanicals as complex mixtures (Figure 2). The assays included in the current battery were suggested and selected by experts in cardiac *in vitro* tools, most with experience from initiatives like the Comprehensive *In vitro* Proarrhythmia Assay (CiPA), which seeks to redefine preclinical cardiac safety assessment for new drugs. When selecting assays, experts identified tools that were established and reproducible, relevant for key mechanisms associated with botanical-induced cardiotoxicity, and accessible.

For assay qualification in cardiotoxicity studies, sematilide, quinidine, and procainamide are appropriate positive controls because they produce reproducible, mechanistically informative perturbations of cardiac electrophysiology: sematilide is a class III IKr blocker that prolongs action potential duration, while quinidine and procainamide are class Ia agents that primarily inhibit fast Na^+^ current with additional repolarization effects that lengthen QT (the time between the start of ventricular depolarization and the end of repolarization; prolongation of QT interval can indicate an increased risk for arrhythmia) and can unmask proarrhythmic liability; inclusion of such mechanistically diverse reference drugs is consistent with best-practice recommendations for repolarization studies in human stem cell-derived cardiomyocyte assays (Gintant et al., 2020).

### 2.1 *In vitro* cell lines

Most of the assays selected for this effort utilized human-induced pluripotent stem cell-derived cardiomyocytes (hiPSC-CM). Some safety pharmacology screen assays use other cell lines, including human embryonic kidney (HEK)293. Although hiPSC-CMs often display fetal-like phenotypes [e.g., low expression of inward rectifying potassium channels or a more depolarized resting membrane potential (Sala et al., 2017a; Garg et al., 2018; Savoji et al., 2019)], they express all major cardiac ion channels found in adult cardiomyocytes (Garg et al., 2018; Karakikes et al., 2015). Furthermore, hiPSC-CMs are widely being evaluated as an alternative model for cardiac safety assessment, such as in the Comprehensive *In vitro* Proarrhythmia Assay (CiPA) initiative (Blinova et al., 2018).

Although hiPSC-CM assays have been extensively used to test drugs (Ma et al., 2024; Malan et al., 2016; Yanagida et al., 2024; Yanagida and Kanda, 2024; Zeng et al., 2019; Zhao et al., 2018) and to inform authorities of cardiac safety in investigational new drug applications (Yang et al., 2022), the use of these assays to detect cardiotoxicity induced by complex mixtures such as environmental samples or plant extracts has not been extensively reported and still requires testing and evaluation (Burnett et al., 2021).

### 2.2 Microelectrode array

**FIGURE 3:**
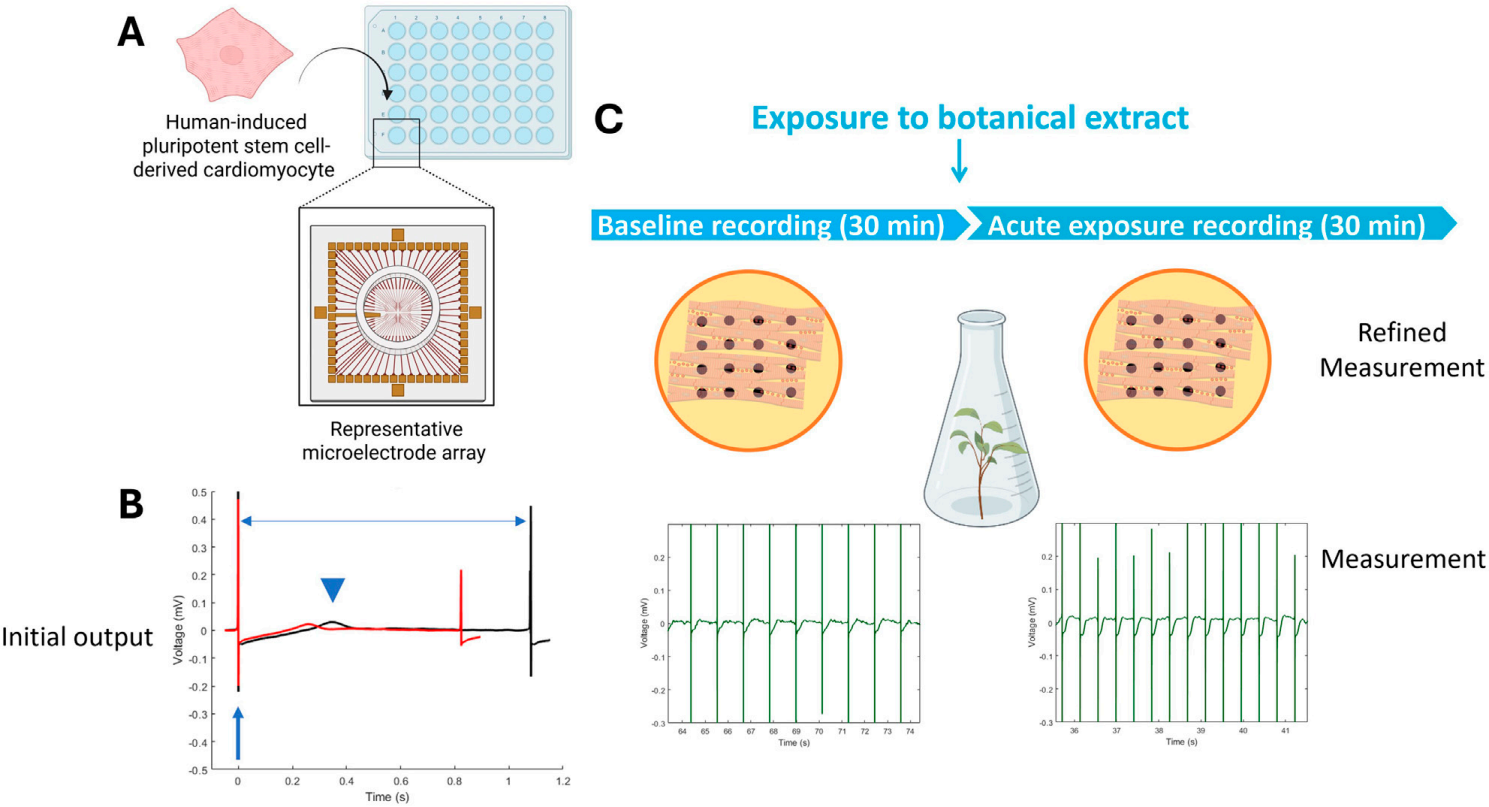
Microelectrode Array (MEA) recordings rely on 48-well or 96-well plates that have 8–16 electrodes on the bottom of each well, on top of which cardiomyocytes can be cultured **(A)**. Cardiac function is recorded as an electrical signal with distinct features, such as the depolarization peak (arrow), repolarization (arrowhead), and beat period (horizontal arrow) that can change between baseline (black) and exposure (red) **(B)**. The cardiac-modulatory potential of botanical extracts can be assessed by comparing activity before (i.e., baseline recording) and after acute exposure **(C)**. Figure created in BioRender.

Microelectrode array (MEA) recordings provide a non-invasive, kinetic method to study cardiomyocyte function by recording extracellular field potentials in hiPSC-CM monolayers (Figure 3) (Sala et al., 2017b). This approach assesses the impact on multiple cardiac targets, including ion channels, using spontaneously active hiPSC-CMs cultured on MEAs as a physiologically relevant model for cardiotoxicity assessment (Sala et al., 2017a). The field potentials recorded with the MEA correlate to intracellular membrane action potentials typically measured using patch-clamp recording. Moreover, drug-induced effects on field potentials correlate well with standard functional cardiac electrophysiology methods (Harris et al., 2013).

While MEAs provide useful functional readouts, they cannot identify precise molecular targets, so complementary assays are often needed for mechanistic clarity. They are also only moderate in throughput compared to simpler screening methods, which limits their use for very large libraries. Finally, although MEA parameters map to electrocardiogram (ECG)-like features, translating these signals into clinical outcomes remains uncertain without considering human absorption, metabolism, and exposure. The extracellular field potential recordings allow for the investigation of several functional parameters, including beat rate, field potential duration, and spike amplitude. These parameters closely resemble the heart rate, QT interval, and QRS amplitude (the height of a wave or deflection on an ECG graph that represents ventricular depolarization; important in the diagnosis of ventricular hypertrophy) in an ECG, thereby enabling translation of *in vitro* findings to *in vivo* effects. Consequently, hiPSC-CMs cultured on MEA recordings hold great potential to study drug-induced electrophysiological alterations, torsadogenic potential, and arrhythmias (Andrysiak et al., 2021; Asakura et al., 2015; Blinova et al., 2018; Kanda et al., 2018; Satsuka and Kanda, 2020; Takasuna et al., 2020; Zhao et al., 2022).

Recently, hiPSC-CMs monolayers cultured on MEAs have been used to detect proarrhythmic effects of plant extracts (*Evodia rutaecarpa* preparations containing different amounts of the human ether-a-go-go related gene (hERG) inhibitors dehydroevodiamine and hortiamine). Using this approach, it was shown that the extracts dose-dependently prolonged the field potential duration (Baltov et al., 2023). This recent study highlights the applicability of hiPSC-CMs cultured on MEAs for predicting human cardiotoxicity by plant extracts.

### 2.3 Optical mapping of voltage changes

**FIGURE 4:**
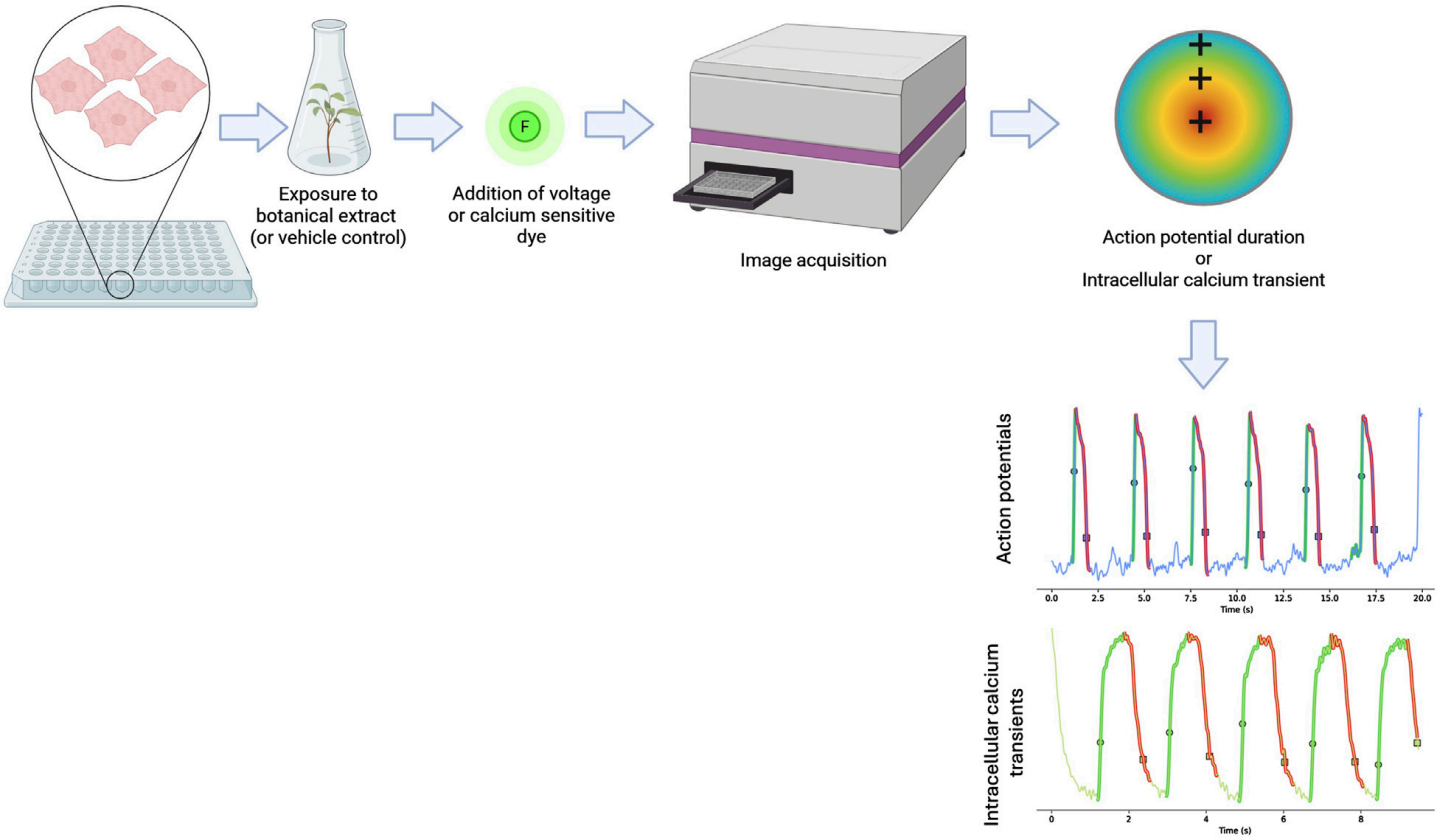
Schematic diagram showing how optical mapping is conducted. Human-induced pluripotent stem cell-derived cardiomyocytes (hiPSC-CMs) are plated onto well plates and exposed to a botanical extract or a solvent control for a period of time before a voltage or calcium-sensitive dye is added. The activation of the dye by the depolarizing hiPSC-CMs is then measured and used to measure action potentials and intracellular calcium transients to capture key electrophysiological parameters. Figure created in BioRender.

Voltage-sensitive dyes allow for the assessment of membrane potential changes through optical imaging. These dyes (such as electrochromism-based styryl dyes and dyes that rely on photo-induced electron transfer) bind to the external surface of cell membranes without disrupting function, and their fluorescence intensity varies with changes in membrane potential (Chemla and Chavane, 2010; O’Shea et al., 2020). This method enables the evaluation of action potential waveform and duration in hiPSC-CMs, providing insights relevant to QT interval assessment (Figure 4).

Optical mapping for investigating drug-induced changes in action potentials has been validated in multicenter and international consortium studies (Blinova et al., 2018; 2017; Kanda et al., 2018). While MEA have been widely used for arrhythmogenic assessment, studies indicate that optical mapping can provide higher spatiotemporal resolution (Gintant et al., 2020). Optical mapping can capture key electrophysiological parameters such as action potential duration, heterogeneity, and conduction velocity using high-resolution charged-coupled device cameras. Incorporating conduction velocity into arrhythmogenesis risk assessment models has improved predictive accuracy (da Rocha et al., 2020). Given its ability to detect subtle electrophysiological alterations, optical mapping has been widely applied in pharmaceutical screening (Blinova et al., 2018; Gunawan et al., 2021; Strauss et al., 2019).

Optical mapping can be affected by dye-related artifacts such as photobleaching or cytotoxicity (O’Shea et al., 2020). While it provides detailed information on action potentials, it does not identify specific molecular targets, and the use of simplified monolayers limits its physiological relevance compared to intact cardiac tissue. While extensively used for single chemicals, the applicability of optical mapping for botanicals and complex chemical mixtures remains largely unexplored. Determining whether this method can reliably assess their electrophysiological effects is an area of ongoing investigation.

### 2.4 Optical mapping of intracellular calcium transients

Changes in intracellular calcium transients offer critical insights into cardiomyocyte function, as calcium-handling abnormalities are associated with arrhythmogenesis, contractile dysfunction, and other cardiac pathologies. Optical mapping of calcium transients is conducted in a similar manner as the optical mapping of voltage sensitive dyes, with the primary difference being the type of dye used (Figure 4). Fluorescent dyes used for the mapping of intracellular calcium transients react to the presence of calcium ions released from the plated cells. Numerous studies have used different optical imaging modalities to investigate calcium transient alterations caused by mutations, acquired diseases, and drug exposure (Allan et al., 2023; Davis et al., 2021; Di Pasquale et al., 2013; Kim J. J. et al., 2015; Lee et al., 2012; Monteiro Da Rocha et al., 2023; Shafaattalab et al., 2019).

In hiPSC-CMs, optical mapping of calcium transients has been used to evaluate responses to β-adrenergic stimulation and calcium channel blockers. For instance, the chronotropic, inotropic, and lusitropic effects of isoproterenol treatment are easily observed using this approach (Allan et al., 2023; Davis et al., 2021; Monteiro Da Rocha et al., 2023; Pioner et al., 2019). Additionally, hiPSC-CMs exhibit expected responses to calcium channel blockers, demonstrating the physiological relevance of this system (Bedut et al., 2016; da Rocha et al., 2020). Beyond pharmacological applications, calcium transient mapping has provided insights into conduction abnormalities, including unidirectional conduction block, gap junction uncoupling, ischemia, alternans, and anisotropy in cardiac monolayers (Himel et al., 2012).

Calcium-sensitive indicators can buffer calcium or introduce toxicity, which may alter cell physiology. Because calcium transients are downstream of voltage changes, they provide an indirect view of excitability, and their slower kinetics can obscure rapid electrical events. In addition, simplified monolayer cultures lack the structural complexity of intact myocardium, which limits translation to *in vivo* outcomes.

Given the potential for botanicals to influence calcium handling, optical mapping could be a valuable tool for assessing their cardiotropic effects. However, the inherent complexity of botanical extracts may introduce challenges in identifying specific bioactive components and interpreting concentration-response relationships. Further studies are needed to determine whether this method can effectively capture the cardiophysiological impact of botanical mixtures.

### 2.5 Mitochondrial function

**FIGURE 5:**
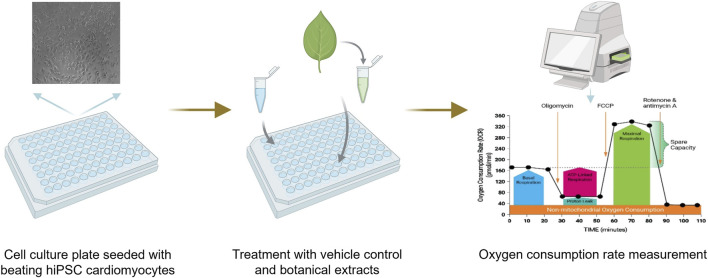
Schematic diagram showing how mitochondrial dysfunction is measured in the seahorse assay. Beating human-induced pluripotent stem cell-derived cardiomyocytes (hiPSC-CM) are plated and then exposed to a botanical extract or vehicle control. After exposure, oxygen consumption rates are measured to assess potential mitochondrial dysfunction.

Mitochondrial dysfunction is a critical factor in cardiotoxicity, contributing to impaired ATP production, increased oxidative stress, and cell death, all of which can compromise cardiac function (Brown et al., 2017). The Seahorse Cell Mito Stress Test (Agilent Technologies) is a widely used assay that assesses mitochondrial function by measuring the oxygen consumption rate (OCR) of live cells in real time (Leung and Chu, 2018). This method utilizes a multi-well plate with built-in injection ports that introduce modulators of cellular respiration, allowing for the dynamic assessment of mitochondrial activity. The assay measures key parameters, including basal respiration, ATP-linked respiration, maximal and spare respiratory capacities, and non-mitochondrial respiration (Figure 5). Specific modulators include oligomycin, which inhibits ATP synthase and reduces OCR; carbonyl cyanide-p-trifluoromethoxyphenylhydrazone (FCCP), an uncoupler that disrupts the mitochondrial membrane potential and increases OCR; and rotenone/antimycin A, which inhibits the electron transport chain and decreases OCR.

Mitochondrial function is particularly important in cardiomyocytes, where ATP production directly supports cardiac contraction and vascular function. Impaired mitochondrial activity can lead to reduced cardiac output and systemic effects on cardiovascular health. The Seahorse assay has been widely applied to evaluate mitochondrial toxicity induced by clinically relevant drugs such as aspirin, doxorubicin, and simvastatin (Holt et al., 2023; Kretzschmar et al., 2021; Kuzyk et al., 2020; Liu et al., 2024). While its use with botanical extracts is less explored, this approach could provide insights into plant-derived compounds that influence mitochondrial function. A key limitation of the assay is that it measures OCR only in cultured cells, not in intact tissues, which may limit physiological relevance (Divakaruni et al., 2014). Additionally, variability in cell adhesion, plating density, and cytoplasmic metabolism can impact reproducibility, necessitating careful optimization of experimental conditions (Brand and Nicholls, 2011; Lange et al., 2012; Luz et al., 2015).

### 2.6 Contractile force

**FIGURE 6:**
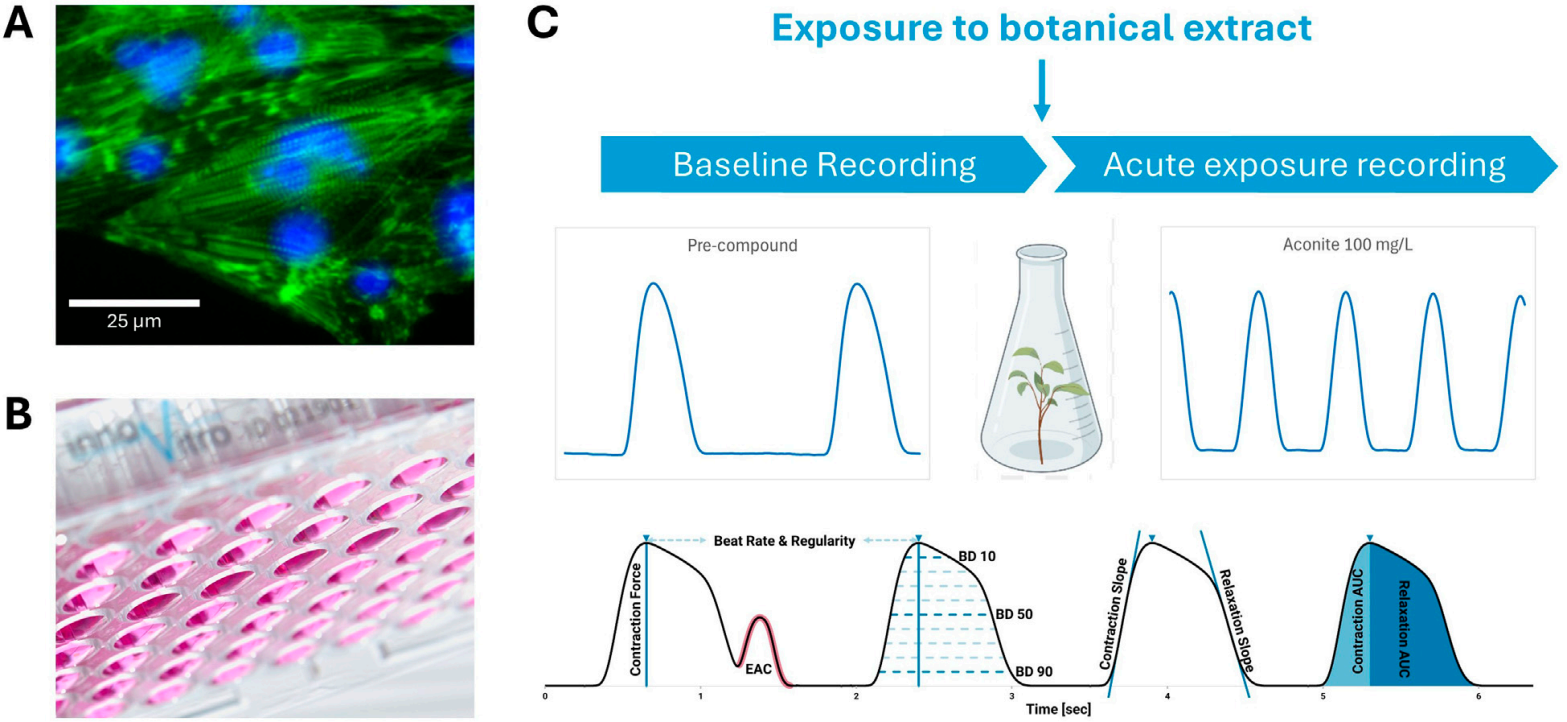
A schematic illustrating the direct measurement of contractile force. Human-induced pluripotent stem cell-derived cardiomyocytes **(A)** cardiomyocytes stained with a combined phalloidin/DAPI (4′,6-diamidino-2-phenylindole) for visualizing F-actin and nuclei, respectively are plated on freely swinging polydimethylsiloxane membranes **(B)**, which move when the plated cardiomyocytes contract. A baseline recording is taken **(C)** before the same cells are exposed to a botanical extract or a vehicle control. Then another recording is taken and compared to the baseline to evaluate changes in the contractile force of the cardiomyocytes **(C)**. EAC, early after contraction.

The *in vitro* assessment of cardiotoxicity often involves measuring contractile force, which can be performed either directly or through impedance-based techniques. Direct measurement of contractile force typically utilizes isolated cardiomyocytes or tissue constructs, where mechanical sensors detect the force generated during cell contraction (Figure 6). This method provides precise, real-time data on the contractile properties of heart cells, crucial for evaluating the impact of drugs or compounds on cardiac function.

Alternatively, impedance measurements offer a non-invasive approach to assess contractility indirectly by detecting changes in electrical resistance as cardiomyocytes contract and relax. Electrodes embedded in the culture substrate continuously monitor these impedance fluctuations, which correspond to cell movement and morphology changes. This technique enables high-throughput screening of cardiotoxic effects and provides dynamic information on beat rate, amplitude, and rhythmicity.

The FLEXcyte 96 technology (Nanion Technologies GmbH, Munich, Germany) integrates both contractile force measurement and impedance-based assessment in a 96-well format, offering a physiologically relevant mechanical environment (Gossmann et al., 2020). In this system, hiPSC-CMs are seeded on freely swinging polydimethylsiloxane (PDMS) membranes (innoVitro GmbH, Juelich, Germany), which bend under the weight of the culture medium (Figure 6) (Gossmann et al., 2016). As the cardiomyocytes contract, the membranes lift, and this movement is recorded. Contractile parameters—including force of contraction, beat duration, beat frequency, contraction and relaxation velocity, and area under the curve—are derived using Laplace’s Law (Gossmann et al., 2016). Additionally, impedance monitoring in this system detects changes in cellular adhesion, morphology, and conductivity, offering an integrated assessment of cardiomyocyte functionality. This approach has been used to evaluate the potential cardiotoxicity of single chemicals including G protein-coupled receptor (GPCR) agonists, calcium channel agonist, an hyperpolarization-activated cyclic nucleotide-gated (HCN) channel antagonist and potassium channel antagonists (Lickiss et al., 2024); however, there is a need for additional work to evaluate the suitability of the method for use with complex mixtures such as botanical extracts.

Contractility assays may miss subtle electrophysiological changes that do not translate into altered contraction, and they provide only an indirect view of ion channel activity. Immature sarcomere organization in iPSC-derived cardiomyocytes can further limit fidelity to adult myocardium. In addition, both monolayer and engineered tissue models lack systemic inputs such as neurohormonal regulation and hemodynamic load, which are key determinants of contractile performance *in vivo*.

### 2.7 Safety pharmacology screen

Safety pharmacology screens are widely used to identify potential adverse effects on key organ systems, with the heart being a particular area of concern. These screens bring together complementary assays that capture different aspects of cardiomyocyte function, such as excitability, calcium handling, and contractility. For the purposes of this strategy paper, we reference one version of a safety pharmacology screen that illustrates how such assays can be combined to provide an integrated view of cardiac activity (Table 1).

**TABLE 1:** A list of the assays evaluated in the selected safety pharmacology screen as well as the specific mechanisms the assay assessed and the information that could be derived from the measurements.

Assay name	Receptor/Transporter/Enzyme	Measurement
FLIPR Calcium Assay	Adrenergic alpha1a	Increase in intracellular calcium levels, which is measured using a calcium-specific dye via fluorescence
	Dopamine 1	
	Histamine 1	
	Muscarinic 1	
	Muscarinic 3	
	Serotonin 2b	
Beta Arrestin Assays	Adrenergic beta 2	Impact of beta arrestin activation on cardiomyocyte contractility, relaxation, and survival. Enzyme fragment complementation, where β-arrestin recruitment to the G protein-coupled receptor (GPCR) forms an active β-galactosidase enzyme, generating luminescence upon substrate addition.
	Cannabinoid 1	
	Mu opioid	
Amine Transporter Assays	Norepinephrine	Inhibition of transporters by measuring real time uptake of a dye labeled amine via fluorescence
	Dopamine	
	Serotonin	
Human ether-a-go-go related gene (hERG) Binding Assay	hERG	Binding activity using hERG-expressing human embryonic kidney (HEK) cells. Cells prepared with Cy3B-labeled ligand, and fluorescence polarization measurements determine binding inhibitory constant (Ki) values.
Ion Channel Profiling	hERG	Ionic currents with automated patch clamp. Activation and Inhibition measured
	Nav1.5	
	Cav1.2	
GABA Gamma-aminobutyric acid (GABA) Patch Clamp Assay	GABA A receptor (α1β2γ2)	Chloride currents measured using whole-cell patch clamp in GABA_A receptor (α1β2γ2)-expressing cells. Changes in current amplitude and kinetics assess receptor activation, inhibition, or modulation.
Phosphodiesterase (PDE) Assays	PDE 3A1, 4D3, 5A1	Intracellular levels of cyclic adenosine monophosphate (cAMP) using scintillation proximity assay (SPA) beads in 384-well plates. Radioactive counts assess enzymatic activity.
Bromodomain-Containing Protein 4 (BRD4) Binding Assay	BRD4	Interaction with the bromodomain of BRD4, potentially disrupting its function in cellular processes like gene transcription. Fluorescence polarization assay with Cy5-labelled probe binding to BRD4 protein
Acetylcholinesterase Assay	Acetylcholinesterase	Enzyme activity using Ellman’s method; thiocholine formation induces color change read at 405 nm, proportional to enzyme activity.

While widely used in the pharmaceutical industry, safety pharmacology assays often focus on single targets, such as hERG, which can miss broader pharmacological effects. In addition, these assays do not capture absorption, metabolism, or systemic distribution, which are critical for translating findings to human exposure.

## 3 Botanical case studies

To evaluate the above-described screening assays, the Cardiotoxicity Working Group nominated botanicals to be used as demonstrative case studies. Of those nominated (Table 2), several are not typically taken as dietary supplements (e.g., aconite, oleander, and comfrey), but their known toxicity makes them useful case studies for the assays. In addition to the botanicals specifically selected by the Cardiotoxicity Working Group, other botanicals were selected as case studies by other working groups and may have limited or no data with respect to cardiotoxicity. The botanicals selected by the BSC were based on the existing literature, including data in humans (e.g., adverse event reporting or clinical trials), animal data (e.g., rodent studies), or mechanistic studies (e.g., cell-based studies), and often a combination of these data types. Prioritization for inclusion in the study was based on availability of chemical analyses data (e.g., constituent identification and qualification). These data are described below in a nonsystematic review. It should be noted that botanical safety information is varied with respect to information reported and quality (Patel et al., 2023). The selected botanicals have been sourced and chemically analyzed to verify the authentication of the test materials (National Toxicology Program, 2023; Waidyanatha et al., 2024). Table 3 provides an overview of available cardiotoxicity data for each botanical case study.

**TABLE 2:** List of botanical extracts used in the BSC, including their standardized common and scientific names, Distributed Structure-Searchable Toxicity substance identifier (DTXSID), and part(s) of the plant used to derive the extract. Botanicals with suspected cardiotoxicity are bold. Table is modified from Waidyanatha et al. (2024).

Standardized common name	Scientific name	DTXSID^a^	Plant part(s)	Details
Aconite	*Aconitum napellus* L., Ranunculaceae	DTXSID701061676	Mixed parts	95% ethanol extract
Aristolochia fangchi	*Aristolochia fangchi* Y. C. Wu ex L. D. Chou and S. M. Hwang, Aristolochiaceae	DTXSID201349132	Root	95% ethanol extract
Ashwagandha	*Withania somnifera* (L.) Dunal, Solanaceae	DTXSID201042372	Root	Ethanol: Water ∼15:1) extract; 1.56% total withanolides
Asian Ginseng	*Panax ginseng* C.A.Mey., Araliaceae	DTXSID1023780	Root	Ginseng Dry Extract 4% (Quintozene free); 4.6% total ginsenosides
Blue cohosh	*Caulophyllum thalictroides* (L.) Michx., Berberidaceae	DTXSID401042859	Root and Rhizome	95% ethanol extract
Comfrey	*Symphytum officinale* L., Boraginaceae	DTXSID20274226	Root	95% ethanol extract
Ephedra	*Ephedra sinica Stapf*, Ephedraceae	DTXSID801018482	Aerial Parts	95% ethanol extract
Goldenseal	*Hydrastis canadensis* L., Ranunculaceae	DTXSID40274228	Root and Rhizome	95% ethanol extract
Green tea decaffeinated extract	*Camellia sinensis* (L.) Kuntze, Theaceae	DTXSID0031398	Leaf	Green tea dry decaffeinated extract; 78.5% total catechins
Kava	*Piper methysticum* G. Forst., Piperaceae	DTXSID901018742	Root and Rhizome	95% ethanol extract
Kratom	*Mitragyna speciosa* Korth., Rubiaceae	DTXSID001334842	Leaf	95% ethanol extract
Milk thistle	*Silybum marianum* (L.) Gaertn., Asteraceae	DTXSID8031657	Seed	Milk thistle dry extract; 90.6% of silymarin isomers
Oleander	*Nerium oleander* L., Apocynaceae	DTXSID201042091	Stem	95% ethanol extract
Tripterygium^b^	*Tripterygium wilfordii* Hook.f., Celastraceae	DTXSID301349830	Root	95% ethanol extract
*Usnea* lichen	*Usnea* spp., Parmeliaceae	DTXSID701349537	Whole Lichen	95% ethanol extract
Yohimbe	*Pausinystalia johimbe* (K. Schum.) Pierre, Rubiaceae (currently accepted name *Corynanthe johimb*e K. Schum.)	DTXSID4032291	Bark	95% ethanol extract

^a^ Substance identifier used in Environmental Protection Agency CompTox dashboard (https://www.epa.gov/chemical-research/comptox-chemicals-dashboard). Note, DTXSIDs have been created for all plants but are iteratively added to the EPA site.

^b^ Commonly known as thunder god vine.

**TABLE 3:** Summary of available data types for selected botanicals. This was not a systematic literature search, and the quality of the studies was not evaluated and may vary. Given the complex nature of botanical extracts, the chemical composition of the tested materials included in the review and the extracts prepared by the Consortia may be different due to differences in the source and preparation of the materials.

Standardized common name	Summary with respect to cardioactivity	Cardioactivity and evidence type(s)
Aconite	Known to induce cardiac effects	• Reports of rhythmic effects in humans (Chen et al., 2012; Lin et al., 2004) and animal studies (Amran et al., 2004; Bartosova et al., 2007; Ohno et al., 1992; Wu et al., 2017) • Effects on repolarization *in vitro* (Honerajger and Meissner, 1983; Nilius et al., 1986; Wu et al., 2017)
Aristolochia fangchi	Unknown	
Ashwagandha	Unknown	
Asian ginseng	Not expected to induce cardiac effects	
Blue cohosh	Unknown	
Comfrey	Unknown	
Ephedra	Known to induce cardiac effects	• Reports of rhythmic effects in humans (Andraws et al., 2005; Fleming, 2000; Haller et al., 2002) • Reports of vasoactive effects in humans (Andraws et al., 2005; Haller et al., 2002) • Reports of myocardial injury in animal studies (Dunnick et al., 2007)
Green Tea	Not expected to induce cardiac effects	
Goldenseal	Unknown	
Kava	Unknown	
Kratom	Known to induce cardiac effects	• Reports of rhythmic effects in humans (Abdullah et al., 2021; Abdullah and Singh, 2021; Anwar et al., 2016; Eggleston et al., 2019; Graves et al., 2021; Grundmann, 2017; Kruegel and Grundmann, 2018; Sethi et al., 2020) • Effects on repolarization *in vitro* (Tay et al., 2016)
Milk thistle	Not expected to induce cardiac effects	
Oleander	Known to induce cardiac effects	• Reports of rhythmic effects in humans (Farkhondeh et al., 2020) • Reports of rhythmic effects in animals (Botelho et al., 2017; Clark et al., 1991)
Tripterygium	Known to induce cardiac effects	• Case reports in humans (Chou et al., 1995; Zhang Q. et al., 2016) • Effects on ion channels *in vitro* (Xu et al., 2023; Zhao et al., 2019)
Usnea lichen	Unknown	
Yohimbe	Known to induce cardiac effects	• Case reports in humans (Anderson et al., 2013; Giampreti et al., 2009) • Reports of rhythmic and vasoactive effects in humans (Linden et al., 1985) • Effects on action potential *in vitro* (Gong et al., 2022) • Reports of cardiotoxicity in animals (Chen et al., 1991; Thomas and Tripathi, 1986)

### 3.1 Aconite

Aconite (*Aconitum napellus* L.), also known as monkshood, is a flowering plant indigenous to West and Central Europe. Traditional medicines prepared from *Aconitum* spp. (including *A. napellus*) are generally prepared from the whole plant or root and purported to have some effects, including anesthetic, analgesic, anti-inflammatory, and antidiarrheal properties (Povšnar et al., 2017). However, there are contemporary case studies documenting fatal or near-fatal poisonings associated with aconite (Pullela et al., 2008; Strzelecki et al., 2010). In cases of overdose, aconite is known to elicit cardiotoxic and neurotoxic effects (Gao et al., 2020; Li et al., 2022). Aconitine [DTXSID4046319] is a toxic alkaloid found in the leaves and roots of the plant.

The total content of aconitine and other toxic alkaloids can be reduced by soaking aconite root in an alkaline solution and boiling; however, even when it is processed using this technique, a poor-quality aconite preparation, when ingested, can cause toxicity, including muscle spasms, paralysis of the respiratory system, and heart rhythm disorders (Chen et al., 2012), and several cases of life-threatening ventricular tachycardia have also been reported (Lin et al., 2004).

Aconitine has been shown to induce ventricular tachycardia and ventricular fibrillation in various animal studies (Amran et al., 2004; Bartosova et al., 2007; Ohno et al., 1992; Wu et al., 2017) and has been demonstrated to act as a late Na^+^ current agonist, leading to delayed repolarization, in isolated cardiomyocytes (Honerajger and Meissner, 1983; Nilius et al., 1986). Additionally, data from hiPSC-CMs indicated that inhibition of the L-type calcium channel currents, as well as increased beating frequency and decreased action potential duration, although no change in late sodium currents, by aconitine could play a key role in the proarrhythmic effects of aconitine in humans (Wu et al., 2017).

Overall, aconite is expected to induce cardiac effects in humans.

### 3.2 Aristolochia fangchi

*Aristolochia fangchi* is a perennial climbing vine native to southeast Asia. It is also known as *fang ji* or *guang fang ji*, and other names that have led to mistaken identity with other plants. In the 1990s in Belgium, over 100 people mistakenly took *A. fangchi* instead of *Stephania tetrandra* for weight loss, leading to kidney damage, severe kidney failure requiring transplants or dialysis in 70 cases, and subsequent cancers or urinary tract diseases. (Debelle et al., 2008; Grady, 2000). *A. fangchi* and other related *Aristolochia* species contain aristolochic acids (such as aristolochic acid I [DTXSID0040969] and II [DTXSID00197166]), which are acknowledged for their genotoxic and renal toxic properties. This species was singled out for investigation by the BSC due to its genotoxic effects (Debelle et al., 2008; Grady, 2000).

Based on searches in Google Scholar and PubMed as of the publication of this article, there are no studies in humans or rodent models for Aristolochia with respect to cardiotoxicity. One study in zebrafish embryos exposed to aristolochic acid I increased the pericardial area, widened sinus venosus and the bulbus arteriosus (a measurement used to assess cardiac development), and reduced heart size and linearization of both ventricles. However, this could be due to the compound causing genotoxicity or developmental toxicity in the developing embryo (Hu et al., 2024).

While there were effects in the zebrafish embryo study, it is unclear what cardiac effects to anticipate for *A. fangchi* in the select assays.

### 3.3 Ashwagandha

Ashwagandha (*Withania somnifera*), also known as Indian ginseng, is an evergreen shrub indigenous to arid regions of tropical and subtropical zones across Asia, Africa, and the Middle East. It has been used in Ayurveda and other traditional medicinal practices and is often purported to enhance resilience against various stressors (Speers et al., 2021). The selection of ashwagandha root extract by the BSC stemmed from the existing developmental toxicity studies with negative results, and there have been documented instances of hepatotoxicity (Lubarska et al., 2023; Siddiqui et al., 2021). Biologically significant constituents, including ashwagandhanolide [DTXSID301353590], withaferin A [DTXSID10965459], and withanoside I [DTXSID801355610] have been identified in its root (Pal et al., 2012).

Short-term administration of ashwagandha extract (e.g., up to 3 months) may be safe, but there is not enough information on its long-term safety (NCCIH, 2023). A review paper from Brown (2018), reports in a table that there is shock and ventricular tachycardia in case reports related to ashwagandha. This comes from another review which reports a case in a 60-year-old male taking *W. somnifer*a with “VT, ventricular bigemini, LBBB” and a case of a 55-year old that presented with ventricular tachycardia (Dwivedi et al., 2011). The review is on 12 patients treated at the University of Delhi, but no details are given on the proper identification and characterization of the material.

Standardized ashwagandha root extracts (1.5% withanolides) given to rats at a dose of 300 mg/kg were reported to reduce cardiotoxicity induced by doxorubicin and restore several biochemical changes, such as reduction of elevated malondialdehyde levels, catalase, superoxide dismutase activity, calcium content, Bcl-2 protein levels (Hamza et al., 2008).

A review by Wiciński et al. (2024) summarized data for rodents, cell lines, clinical studies, and other models on ashwagandha’s cardiovascular effects, with results from many of the assays indicating that ashwagandha results in lowering inflammation and anti-angiogenic effects.

While cardiotoxic effects from ashwagandha seem unlikely, it is unclear whether ashwagandha will elicit cardiac effects in the selected assays.

### 3.4 Asian ginseng

Asian ginseng (*Panax ginseng*) is a perennial herb characterized by its branched root shape. Asian ginseng is typically cultivated for a span of 6–10 years before being harvested. Originating from regions across Asia, including China and Korea, it has been integral to health-related practices for hundreds of years. According to the National Center for Complementary and Integrative Health (NCCIH), short-term oral usage (up to 6 months) is generally considered safe for most individuals (NCCIH, 2020). The selection of the root extract of this plant by the BSC was based on the absence of adverse effects for various endpoints supported in the literature. Asian ginseng contains ginsenoside saponins. These chemicals have been shown to have some therapeutic effects (Jia et al., 2009; Vijayakumar and Kim, 2023; Wang et al., 2009).

Clinical trials have been conducted using different doses in either healthy subjects or patients with various conditions, including cardiovascular and metabolic diseases (Kim Y.-S. et al., 2015). Single doses up to 800 mg of *P. ginseng* in caffeinated beverages, such as energy drinks, have not been associated with changes in electrocardiogram parameters or increases in heart rate or blood pressure (Shah et al., 2016). Studies in *in vitro* cell cultures and *in vivo* animal models have shown ginseng’s potential cardiovascular benefits through various mechanisms, including antioxidation, modifying vasomotor function, reducing platelet adhesion, influencing ion channels, altering autonomic neurotransmitter release, and improving lipid profiles (Kim, 2012), as well as enhancing mitochondrial respiration capacity and ATP production in cardiomyocytes (Huang et al., 2022).

Overall, Asian ginseng is not expected to cause cardiotoxicity but may have effects on cardiological activity in the select assays.

### 3.5 Blue cohosh

Blue cohosh (*Caulophyllum thalictroides*), not to be confused with black cohosh (*Actaea racemose*), is a flowering plant in the barberry family of plants native to eastern North America. Blue cohosh has been used by midwives to help induce labor; however, it has been associated with perinatal stroke, congestive heart failure, and neonatal shock (Datta et al., 2014). It has been used in combination with other botanicals for abortive or contraceptive purposes.

Blue cohosh root contains N-methylcytosine [DTXSID60149949], which has nicotine-like effects (Scott and Chen, 1943), and caulosaponin (no DTXSID or CASRN available), a glycoside that constricts coronary vessels (NICHD, 2006). While there were no extensive studies conducted investigating the effects of blue cohosh on the cardiovascular system, acute exposure to nicotinic and nicotinic-like alkaloids can have cardiovascular effects such as tachycardia and bradycardia (Schep et al., 2009).

Blue cohosh root extract disrupted cardiovascular and craniofacial cartilage development in medaka embryos in a dose and developmental stage-specific manner (Wu et al., 2010).

More research is needed to see if blue cohosh will induce any effects on the heart, especially in adults.

### 3.6 Comfrey

Comfrey (*Symphytum officinale*) is a perennial flowering plant indigenous to Asia and Europe. It has historically been used in wound healing applications. However, it contains known genotoxic compounds, specifically pyrrolizidine alkaloids (PAs) (Schramm et al., 2019). Among the identified PAs in comfrey are lycopsamine [DTXSID60145542] and 7-acetyllycopsamine [DTXSID50223742].

One clinical study monitored heart rate and blood pressure when patients were using a daily topical application that contained comfrey extract and reported no differences between comfrey and placebo (Grube et al., 2007). While there were several cardiovascular-related adverse event reports in the literature where individuals mistakenly consumed foxglove (*Digitalis purpurea*) instead of comfrey (Lin et al., 2010; Vithayathil and Edwards, 2016) there have been no extensive studies conducted on comfrey’s (or its constituents’) effects on the heart.

It is unknown whether comfrey will induce cardiotoxic effects in the selected *in vitro* assays.

### 3.7 Ephedra

Ephedra (*Ephedra sinica*), also known as Ma Huang, is an herbaceous perennial plant native to China (NIDDK, 2012). Constituents of biological interest include alkaloids like ephedrine [DTXSID0022985], pseudoephedrine [DTXSID0023537], and N-methylephedrine [DTXSID401021166] (NIDDK, 2012). In China and India, it has been used to treat various conditions, such as colds, fever, and headache, and has been purported to promote weight loss. Ephedra was used in weight loss supplements; however, adverse effects including cardiovascular effects and neurological effects (e.g., heart attack, seizure, stroke, and death) led to the US Food and Drug Administration (US FDA) banning all dietary supplements containing ephedrine alkaloids from being sold in the US in 2004 (US FDA, 2004a; 2004b).

If abused, taken in high dosages, or taken by consumers who have pre-existing cardiovascular diseases, ephedra may contribute to adverse events. There are several deaths reported from an overdose of ephedra. (Gurley et al., 1998; Theoharides, 1997). Symptoms of overdose include heart palpitations, extreme nervousness, sweating, enlarged pupils, severe headache, dizziness, dyspnea, elevated body temperature, and, ultimately, death (Fleming, 2000).

Reports have suggested that ephedra alkaloids taken at well-controlled doses showed cardiovascular side effects such as increased blood pressure, heart rate, and mild palpitations (Andraws et al., 2005). In a single-dose study in humans, the biological effects of an oral dose of an ephedrine alkaloids supplement included central nervous system stimulation, peripheral vasoconstriction, elevation of blood pressure, bronchodilation, and cardiac stimulation (Haller et al., 2002). A study investigating powdered ephedra in humans reported increased pulse rate, decreased intestinal tone and motility, mydriasis, and tachycardia (White et al., 1997). There is evidence that potential interactions of orally ingested ephedrine alkaloids (mainly ephedrine and/or pseudoephedrine, not necessarily the herb ephedra itself) with cardiac glycosides or halothane can cause arrhythmia (Brinker, 2001).

Weight loss supplements containing ephedra and caffeine have been purported to increase weight loss in individuals when compared to a placebo (Boozer et al., 2002); however, the combination of ephedra or ephedrine with caffeine enhanced the cardiotoxicity over that with the herbal medicine or the active ingredient alone (Dunnick et al., 2007). In a rodent model, cardiotoxicity included hemorrhage, necrosis, and degeneration in the ventricles or interventricular septum within 2–4 h after treatment with ephedra extract/caffeine or ephedrine/caffeine (Dunnick et al., 2007).

Based on the adverse event reports associated with ephedra consumption, ephedra is anticipated to induce an effect in the selected assays for assessing cardiotoxicity.

### 3.8 Goldenseal

Goldenseal (*Hydrastis canadensis*) is a flowering, herbaceous, perennial native to the eastern United States and southeastern Canada. Traditionally, goldenseal has been purported to treat skin disorders, digestive issues, urinary tract infections, and more. However, the NCCIH says “the scientific evidence does not support the use of goldenseal for any health-related purpose.” (NCCIH, 2021). Additionally, goldenseal is known to induce botanical-drug interactions, which could affect people taking medicines for heart conditions. Some of its known constituents include berberine [DTXSID9043857] and hydrastine [DTXSID9025409]. Per NCCIH, “Berberine … has been studied for heart failure, diarrhea, infections, and other health conditions” (NCCIH, 2021).

The National Toxicology Program (NTP) completed feed studies in rats and mice for goldenseal root powder for 2 weeks, 3 months, and 2 years. In the 2-year study, there was increased liver cancer in the high-dose male and female rats and the high-dose male mice. The 2-year NTP study found that goldenseal extract may reduce the background level of cardiomyopathy in F344/N rats (National Toxicology Program, 2010). Berberine has been reported to have both cardiotoxic and cardioprotective effects (Wang et al., 2023) and some studies point to anti-inflammatory properties and potential cardioprotective effects (for review, see Mandal et al., 2020).

Whether goldenseal will induce cardiac effects in the selected *in vitro* assays is unclear.

### 3.9 Green tea dry decaffeinated extract

Green tea (*Camellia sinensis*) is one of the most common beverages in the world and has numerous studies purporting various benefits (NICCIH, 2020). As a steeped beverage with natural levels of caffeine, it is believed to be safe to drink up to eight cups per day. The polyphenols in green tea may account for up to 30% of its dry weight. Most of these polyphenols are catechins, with one of the major polyphenol monomers being epigallocatechin gallate (EGCG [DTXSID1029889]) (Lin et al., 2003).

Several completed clinical phase I-III trials on green tea extracts and/or catechins demonstrated the bioavailability, safety, and effectiveness in modulating clinical and biological markers associated with cancer and non-cancer endpoints. While the tea is considered safe, the BSC focused on a decaffeinated concentrated green tea extract containing high amounts of catechins, similar to extracts commonly used as dietary supplements (National Toxicology Program, 2016). In this project, the Hepatotoxicity Working Group selected decaffeinated concentrated green tea extract due to adverse event reports and by the Genotoxicity Working Group due to its previous evaluation by NTP (National Toxicology Program, 2016). Some data have indicated that higher doses of green tea extracts and/or catechins (e.g., higher than 800 mg EGCG/day) may be associated with moderate to severe abnormalities in liver function (Wu et al., 2011; Yu et al., 2017).

Some studies assessed the cardiotoxic or cardioprotective potential of green tea extracts. One study in healthy human volunteers with different genotypes involved in the inactivation of various catechol-containing chemicals (low vs high activity groups) suggested that green tea extract may have a beneficial effect on small vessel tone in the low activity group (Miller et al., 2012). In animals, many studies report green tea to have cardioprotective effects (Cheng et al., 2016; Ibrahim et al., 2019; Khan et al., 2014; Wang et al., 2016).

Overall, it is unlikely green tea will produce cardiotoxic effects, but it may induce cardiological activity in the select assays.

### 3.10 Kava

Kava (*Piper methysticum*) is a perennial shrub of the pepper family native to the Pacific Islands. The aqueous extracts of kava roots and rhizome have been historically used as a ceremonial beverage but are modernly used as an herbal product purported to have anti-anxiety and pain relief effects (Norton and Ruze, 1994). The BSC Hepatotoxicity working group selected Kava due to adverse event reports associated with kava consumption starting in the 1990s (Soares et al., 2022).

Many lactones have been identified in kava (Soares et al., 2022) including kavain [DTXSID5033595] and 7,8-dihydrokavain [DTXSID101018162]. On average, the kavalactones account for 3%–20% of the dry weight of the kava root (Chua et al., 2016; Sarris et al., 2011).

The NTP conducted 2-week, 3-month, and 2-year studies of kava extract in rats and mice, finding increased liver cancer rates in both male and female mice, higher incidences of liver lesions in both male and female rats, and a slight increase in testicular tumors in male rats. (National Toxicology Program, 2012). There were no reported effects on the cardiovascular system in any of the groups (National Toxicology Program, 2012).

While hepatotoxicity can be considered the main health concern, other reversible (upon interruption of the ingestion or treatment) reported side effects are kava dermopathy, depressant effect along with its anxiolytic properties, drug interaction, gastrointestinal discomfort, nausea, headaches, memory problems, and tremor (Soares et al., 2022). Tachycardia and electrocardiogram abnormalities (tall P waves) have also been reported in heavy kava users (Mathews et al., 1988). One study suggested kava was able to improve reflex vagal control of heart rate in humans with anxiety disorder (Watkins et al., 2001).

Kavain was shown to have an antithrombotic effect *in vitro* on human platelets (Gleitz et al., 1997).

It is unclear whether kava will have an expected cardiac effect in the select *in vitro* assays due to the mix of available scientific literature.

### 3.11 Kratom

Kratom (*Mitragyna speciosa*) is a tropical evergreen tree native to Southeast Asia. More than 40 alkaloids have been identified in kratom extracts, with three of them, mitragynine [DTXSID701032140], corynantheidine (CASRN 23407-35-4), and 7-hydroxymitragynine [DTXSID20903988] known to have pharmacological effects (Meireles et al., 2019). Mitragynine and 7-hydroxymitragynine act on the µ-opioid receptor (Alford et al., 2025; Babu et al., 2008), which has increasingly led to kratom being taken to treat pain and opioid withdrawal without consultation from healthcare providers (Garcia-Romeu et al., 2020). The US FDA has warned that kratom users could experience serious adverse events, including liver toxicity, seizures, and substance use disorder, and ultimately concluded that there is inadequate information regarding kratom to provide reasonable assurance that it does not present a significant or unreasonable risk of illness or injury and, therefore, cannot be legally marketed as a dietary supplement and cannot be lawfully added to conventional foods (Office of the Commissioner, 2023).

There are adverse effect reports resulting from kratom use, including one of a young man who consumed both kratom and Adderall and was found to have a small area of hemorrhage in his brain (Castillo et al., 2017), and others have reported addiction (Vento et al., 2021) and death (McIntyre et al., 2015). Kratom can also cause tachycardia and changes in blood pressure, (Eggleston et al., 2019; Grundmann, 2017; Kruegel and Grundmann, 2018; Sethi et al., 2020). A Poison Control Center report on 3,484 kratom exposures in the US from 2014 to 2019, primarily involving single-substance oral exposures in older adults, found that 45% of cases included cardiovascular effects like hypertension and tachycardia. (Graves et al., 2021). According to an earlier Poison Control Center’s report for kratom exposures between 2010 and 2014, the reported signs and symptoms included tachycardia (25%) and hypertension (12%) (Anwar et al., 2016).

One observational study reported that regular kratom use was associated with at least an 8-fold increase in the odds of individuals presenting with sinus tachycardia when compared with non-kratom users. However, there was no difference in the odds of having other ECG abnormalities (Abdullah et al., 2021). In this study, kratom use was associated with an increased probability of borderline QT interval (QTc) but not prolonged QTc.

In regular kratom users, a study found that higher serum mitragynine levels (at least 9.6 mg/L) were associated with prolongation of the QT interval, and such effects were found to be dose-dependent (Abdullah and Singh, 2021). A mechanistic study showed that mitragynine did not affect the hERG expression at the transcriptional level but inhibited the protein expression (Tay et al., 2016). The hERG tail currents following depolarization pulses were also inhibited by mitragynine (IC_50_ value of 1.62 μM in the hERG-transfected HEK293 cells). In addition, mitragynine inhibited the acetylcholine-activated potassium current through G protein-coupled inwardly rectifying potassium (GIRK) channels (IC_50_ value of 3.32 μM). The study authors concluded that blocking both hERG and GIRK channels may cause additive cardiotoxicity risks (Tay et al., 2016).

Overall, we expect kratom to induce cardiac effects in the select *in vitro* assays.

### 3.12 Milk thistle

Milk thistle (*Silybum marianum*) is an annual plant belonging to the Asteraceae family. It originates from Southern Europe, Russia, Asia Minor, and Northern Africa and has been naturalized in North and South America and Australia (Bijak, 2017). Western herbalists and naturopathic physicians utilize milk thistle fruit for its purported aid for digestive issues. Modern formulations claim to help with liver health (Blumenthal and Busse, 1998).

Generally perceived as safe, milk thistle was chosen by the BSC due to its documented lack of toxicity, notably following an NTP 2-year study conducted on rats and mice (National Toxicology Program, 2011) that found no differences in cardiac effects compared to controls in mice or rats. In addition, a few papers in the literature report that milk thistle can counter chemical-induced cardiotoxicity (Ali et al., 2018; Karimian and Mahmoudi, 2024).

Overall, milk thistle is not expected to induce cardiotoxicity.

### 3.13 Oleander

Oleander (*Nerium oleander*) is a flowering subtropical shrub native to the Mediterranean region but cultivated worldwide as an ornamental plant; it is now found in parts of Asia, Australia, and the Southern United States (INCHEM, 1997). The primary constituent of pharmacological interest is oleandrin [DTXSID40861950], a glycoside. Oleandrin has been used in ethno-phytomedicine as a treatment for a broad spectrum of diseases, including asthma, eczema, and ringworm (Zhai et al., 2022). However, it needs to be noted that oleander is a poison when ingested in sufficient amounts. This has occurred in livestock (Galey et al., 1996) pets, and humans (both intentionally and unintentionally) (Langford and Boor, 1996).

Oleander extracts contain oleandrin and other cardiac glycosides, which can inhibit the activity of Na+/K + ATPase, leading to hyperkalemia. Cardiac glycosides were initially used in the treatment of congestive heart failure and other conditions (Digitalis Investigation Group, 1997). The cardiotoxic effects include hemorrhage, necrosis, arrhythmia, sinus bradycardia, and a prolonged P-R interval for ECG recordings (Farkhondeh et al., 2020).

Oleander has been tested using animal models. In mice and rats, there were elevated troponin levels and indications of hyperemia and hemorrhage (Khordadmehr and Nazifi, 2018). In guinea pigs, there were signs of arrhythmias due to Na+/K+ pump inhibition (Botelho et al., 2017) and in dogs, there were induced dysrhythmias (Clark et al., 1991).

Overall, oleander is known to have cardiac effects and is expected to elicit effects in the selected assays.

### 3.14 Tripterygium

Tripterygium (*Tripterygium wilfordii*), also known as Thunder God Vine, has historically been utilized in traditional Chinese medicine to purportedly treat inflammatory and autoimmune disorders (Dinesh and Rasool, 2019). The root extracts of the plant contain triptolide [DTXSID5041144] and celastrol [DTXSID2040993] as major constituents (Liu et al., 2020).

There are reported overdose cases for tripterygium and other adverse event reports in humans (Chou et al., 1995; Zhang Q. et al., 2016). One review highlighted various toxicities associated with tripterygium, including intestinal, liver, kidney, and other endpoints (Ru et al., 2019). Another review reported that 13% of adverse events in the literature of tripterygium were cardiac-related (Zhang C. et al., 2016). There is a documented case of a young man who suffered cardiac damage and intense vomiting, diarrhea, and other severe symptoms after ingesting tripterygium extract: he tragically passed away within 3 days (Chou et al., 1995).

A study using HEK293 cells found that an aqueous crude extract of tripterygium inhibited the amplitude of the hERG current (Zhao et al., 2019). Another *in vitro* study predicted that tripterygium can target voltage-gated sodium channels (Xu et al., 2023).

Other studies have reported that tripterygium can ameliorate damage from other chemicals. This could be due to its pharmacological activities, such as anticancer, anti-inflammation, antifibrosis, and antiatherosclerosis, at low doses below toxic levels, similar to glycosides having therapeutic or toxic effects depending on the dose (Huang et al., 2021; Song et al., 2023).

Overall, we expect tripterygium to induce cardiac effects in the selected assays.

### 3.15 Usnea

*Usnea* lichen, also known as “beard lichen” due to its filamentous strands that grow from tree branches, is found around the world, with over 350 species (National Toxicology Program, 2022). In Chinese herbal medicine, *Usnea* spp. has been used as a purported treatment for many ailments, including headaches, ocular irritation, malaria, and snake bites (Crawford, 2015). The primary constituent of biological interest is usnic acid [DTXSID0040123], which is found in Asian, European, and North American Usnea species (National Toxicology Program, 2022). The sample used by the BSC is wild-sourced in North America.

A 3-month NTP study demonstrated that exposure to an ethanolic extract of *U. barbata* and *U. hirta* containing 60 mg/kg/day (+/−)-usnic acid can be toxic to male and female F344/N NCTR rats, as evidenced by significant weight loss, morbidity, or death after 3 months of exposure (National Toxicology Program, 2022). No evidence of cardiotoxicity was noted in the NTP study. There is one 14-day study that reported the thinning of the cell content in the myocardium and gene expression changes related to oxidative stress in usnic acid-exposed rats, though this could be due to a high dose (100 mg/kg; Yokouchi et al., 2015). Mendonça et al., 2017, the authors report that usnic acid isolated from *Cladonia substellata* impaired myocardial contractility and reduced atrial contraction in guinea pigs. The authors found this effect was related to reduced calcium entry into myocardial cells. Additionally, *in vitro* in isolated cardiomyocytes, exposure to usnic acid caused irreversible cardiac contracture—again related to calcium homeostasis (Mendonça et al., 2017).

Overall, it is unknown if usnea will induce cardiac effects in the selected *in vitro* assays, given the mix of literature.

### 3.16 Yohimbe

Yohimbe (*Corynanthe johimbe*) is an evergreen of the Rubiaceae family native to tropical regions of the African west coast. The bark has traditionally been taken as a purported treatment for fever, leprosy, and cough, as well as for erectile dysfunction, and as an aphrodisiac in West Africa (EFSA Panel on Food Additives, 2013; Lo Faro et al., 2020). More recently, yohimbe has been sold as an aphrodisiac and an athletic performance enhancer (Clark et al., 1984; Grunewald and Bailey, 1993). The indole alkaloid yohimbine [DTXSID9040130] is one of the primary constituents of biological relevance (EFSA Panel on Food Additives, 2013).

There are several adverse effect reports associated with the yohimbe constituent yohimbine, including a male bodybuilder who experienced severe, but reversible, acute effects, including vomiting, loss of consciousness, and seizures after ingesting 5 g of yohimbine (Giampreti et al., 2009). There also have been reports of yohimbine being found at very high blood levels in two individuals who died unexpectedly (Anderson et al., 2013).

Overdose of yohimbine has been linked to causing transient hypertension, accelerated heart rate, and atrial fibrillation (Linden et al., 1985). In a hiPSC-CM model, yohimbine inhibited the frequency and prolonged the duration of spontaneous action potentials by inhibiting sodium and calcium currents (Gong et al., 2022). Yohimbine was identified as an α_2_-adrenoceptor antagonist (Goldberg and Robertson, 1983) and has been used as a model α_2_-adrenoceptor antagonist in animal studies. In cats, yohimbine antagonizes the antiarrhythmic effect of clonidine (α_2_-adrenoceptor agonist) on intravenous acetylstrophanthidin ventricular tachycardia (Chen et al., 1991). In guinea pigs, yohimbine exacerbated the cardiotoxicity of ouabain (a plant-derived toxic substance), which is linked to arrhythmias and cardiac arrest (Thomas and Tripathi, 1986).

Overall, yohimbe is expected to have cardiac effects.

## 4 Conclusions and next steps

Botanicals are used worldwide, and it is critical to ensure their safety, including the potential for them to elicit cardiotoxic effects. To address this need, the cross-sector team of experts from the BSC has identified several NAMs to test their applicability for evaluating botanicals as complex mixtures, using well-characterized, data-rich botanicals as case studies. Assays were selected based on their reproducibility, relevance to key mechanisms, and accessibility. This work aims to equip researchers with tools to better understand cardiotoxicity, prioritize and guide further testing, and deepen our knowledge of botanical products under evaluation by expanding the botanical safety toolkit. It exemplifies a more mechanism-informed approach to botanical safety assessment, shifting away from reliance on traditional animal testing methods towards more predictive, human-relevant models. The next steps of the Consortium involve testing the selected botanical case studies in these assays, analyzing the data, comparing the findings to existing literature, and developing a toolkit of *in vitro* assays for cardiotoxicity assessment.

While this initial work will focus on hazard identification and screening, future efforts could encompass a more exposure-based approach and aid in investigating the predictivity of NAMs for human responses. Critical considerations in the future will include attempts to evaluate the relevance of *in vitro* findings, considering absorption, distribution, and metabolism in humans. *In vitro* to *in vivo* extrapolation using physiologically based pharmacokinetic modeling and simulation could also aid in defining human-relevant concentrations. Advanced complex cellular co-culture models could also provide additional insights into the clinical relevance of toxicity findings and help identify specific effects and mechanisms of action beyond the capabilities of screening-level assays. In addition, exploring how these assays perform with a wider array of botanical extract matrices will help evaluate the domain of applicability for these methods.

This cardiotoxicity-focused effort represents one component of a broader effort by the Botanical Safety Consortium, which also includes ongoing initiatives in genotoxicity, hepatotoxicity, neurotoxicity, reproductive, and dermal toxicity. These novel studies plan to expand the botanical safety toolkit and give researchers tools to better understand cardiotoxicity, prioritize and plan future testing as needed, and better understand the botanical being tested.
